# Research Progress in In Vitro Screening Techniques for Natural Antithrombotic Medicines

**DOI:** 10.3390/ph18020137

**Published:** 2025-01-21

**Authors:** Xinyang Liu, Lu Chen, Lin Li, Yiqi Yan, Han Zhang

**Affiliations:** 1 Institute of Traditional Chinese Medicine, Tianjin University of Traditional Chinese Medicine, Tianjin 301617, China; lxiny1113@126.com (X.L.); lilintcm@126.com (L.L.); 2 Key Laboratory of Pharmacology of Traditional Chinese Medical Formulae, Tianjin University of Traditional Chinese Medicine, Ministry of Education, Tianjin 301617, China; 3 Instrumental Analysis and Research Center, Tianjin University of Traditional Chinese Medicine, Tianjin 301617, China

**Keywords:** anticoagulation, natural medicines, active components, screening techniques, multimodal screening

## Abstract

Natural medicines play an indispensable role in treating thrombotic-related diseases and a thorough investigation of their material basis is crucial for medicine development. The rapid advancement in medicine-active component screening technologies has paved new avenues for studying natural medicines, holding significant theoretical and practical value. This review focuses on the application progress of multimodal screening technologies, including high-throughput screening, chip technology, molecular biology methods, fluorescence sensors, and computational biology, in the screening of anticoagulant medicines. The aim is to provide a reference framework for screening and validating active components in natural medicines. The early application of these technologies can swiftly assess the safety and efficacy of medicines, accelerating the medicine development process and reducing the failure rate in clinical trials. Nonetheless, the overall mechanisms of action of natural medicines and the correlation between their chemical components and thrombotic diseases remain challenging areas that require further in-depth exploration and technological innovation.

## 1. Introduction

Thrombotic disorders, which include arterial, venous, and microvascular thrombosis, frequently manifest in the coronary, cerebral, mesenteric, and lower extremity arteries [1]. The pathogenesis of thrombus formation is a complex interplay of coagulation cascades, platelet activation, and platelet-endothelial interactions. A critical component in this process is protein disulfide isomerase (PDI), which is instrumental in promoting platelet activation and fibrin formation. While current anticoagulant and antiplatelet therapies have established efficacy, they are often accompanied by adverse effects, notably hemorrhagic complications, and may prove insufficient for certain patient populations [2]. Consequently, the pursuit of novel therapeutic agents, particularly the exploration of natural anticoagulant substances, assumes significant importance. Such endeavors hold the promise of mitigating adverse effects while augmenting the therapeutic potency of interventions for thrombotic disorders. Natural medicines, with their unique bioactive components, have become important for treating thrombotic diseases. Modern research has found that natural medicines possess multiple pharmacological effects, including improving hemodynamics, promoting blood circulation [3,4,5], thereby preventing thrombosis [6], reducing inflammatory responses [7], and repairing damaged vascular endothelial cells [8]. Current research on natural medicines primarily focuses on separating natural medicine components, activity tracking methods, and evaluation indicators for individual components [9]. However, there is relatively less basic research on the nature of active ingredient groups, lacking a direction related to the overall characteristics of natural medicines. More attention needs to be paid to the overall mechanisms of action of natural medicines and the correlation studies between natural medicines and diseases.

In the application of in vitro screening technologies for natural medicines, complex medicine component analysis, modern biotechnology, and the integrated application of in vitro anticoagulation models are involved. For instance, multimodal screening technologies such as high-throughput screening, chip technology, molecular biology, fluorescence sensors, and computer-aided medicine design are employed. By integrating various analytical methods, the activity and mechanisms of natural medicine components can be comprehensively assessed. This approach accurately identifies active components within natural medicines and deeply explores the interactions and synergistic effects among these components, providing a scientific basis for medicine development and clinical application. The combined application of these technologies not only improves the precision of natural medicine component screening but also provides new research pathways for revealing the material basis of natural medicines.

## 2. Multi-Level Screening of Active Ingredients in Natural Antithrombotic Medicines

### 2.1. Molecular Level

**Figure 1:**
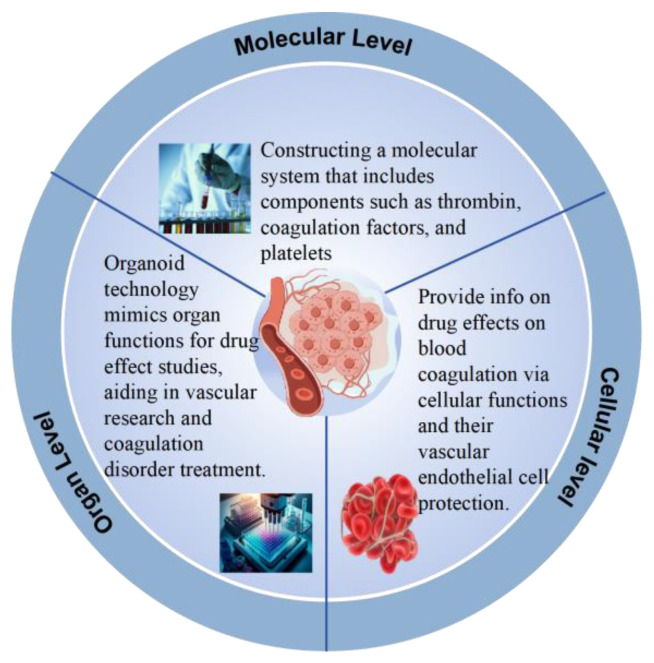
Multi-level screening of active ingredients in natural antithrombotic medicines.

During the multi-tiered screening of natural medicines, The screening at various levels is shown in Figure 1. Researchers utilize contemporary separation technologies to extract individual components from these medicinal sources. Following isolation, these components are then evaluated for their activity using a variety of anticoagulation models [10]. A molecular-level system is established, encompassing crucial blood constituents such as thrombin, coagulation factors, and platelets [11,12,13,14]. By incorporating the natural medicine under investigation into this system, the potential anticoagulant effects can be precisely assessed through the monitoring of the medicine’s specific influence on the coagulation cascade. For example, the anticoagulant effect of medicines can be conveniently and quickly assessed by measuring the extent of prolongation of thrombin time [15]; however, its sensitivity and specificity are relatively low, limiting its use in large-scale and high-throughput screening of basic research on activating blood circulation and removing blood stasis, making it difficult to fully and accurately reveal the scientific essence of treating both disease and symptoms [16]. In addition, methods such as enzyme-linked immunosorbent assay (ELISA) and fluorescence resonance energy transfer technology [17,18] based on specific coagulation or anticoagulation factors are widely used in the screening of medicines for activating blood circulation and removing blood stasis. Jie [19] and his team used ELISA technology to determine the content of related proteins in plasma, exploring the mechanism of action of Xuebijing in coagulation dysfunction in septic rats, confirming the effectiveness of ELISA technology in studying the interaction between medicines and proteins. The high sensitivity and specificity displayed at the molecular level can more accurately assess the anticoagulant activity of medicines.

### 2.2. Cellular Level

The cellular level plays a significant role in the screening of anticoagulant medicines, providing direct information on how medicines affect cellular functions during the blood coagulation process. For example, the effects of medicines on platelet function can be observed through platelet aggregation experiments [20], platelet release reaction tests, etc. Fang [21] and their team confirmed through platelet aggregation experiments and platelet release reaction tests that curcumin plays an important role in regulating the AMPK-V/T-αIIbβ3 signaling pathway to inhibit thrombin-induced platelet aggregation. In addition, experiments at the cellular level can directly assess the protective effects of medicines on vascular endothelial cells. Zhang [22] and their team found that baicalin protects vascular endothelial cells from thrombin-induced cell damage by inhibiting the expression of PAR-1 and its downstream NF-KB activation. Screening at the cellular level can more intuitively demonstrate the relationship between the effects of medicines on the corresponding signaling pathways and their anticoagulant effects.

### 2.3. Organ Level

Organoid technology is an innovative method that simulates the functions of human organs. It constructs models that mimic the structure and function of real human organs through cell culture, microfluidic technology, and bioengineering techniques in a laboratory setting. This technology can more accurately reproduce the mechanisms and effects of medicines within the human body.

Cui [23] developed a novel dual 3D bioprinting technique using PLA and gelatin methacrylate hydrogel to create a vascularized bone biphasic construct. Fluorescently tagged dextrans demonstrated that angiogenic sprouts formed anastomoses with prevascularized networks within the spheroid, preventing core region necrosis. This approach is applicable for vascularizing various spheroids and organoids for medicine testing. Harrison [24] and his team developed a method for creating liver organoids from human pluripotent stem cells (hPSCs) that possess tissue-like vascularization capabilities. This not only mimics the cellular diversity and structure of the liver but also exhibits key liver functions such as medicine metabolism, serum protein production, urea synthesis, and clotting factor production. These organoids can be used for further research on hematopoietic stem cells and tissue-resident hematopoietic cells. The liver organoids cultivated in vitro will have a significant impact on medicine screening, medicine metabolism studies, and research into the mechanisms of various diseases. This achievement provides new strategies for cell therapy, tissue engineering, medicine toxicity assessment, and disease modeling.

## 3. Multi-Modal Screening Technology for Active Ingredients of Natural Antithrombotic Medicines

**Figure 2:**
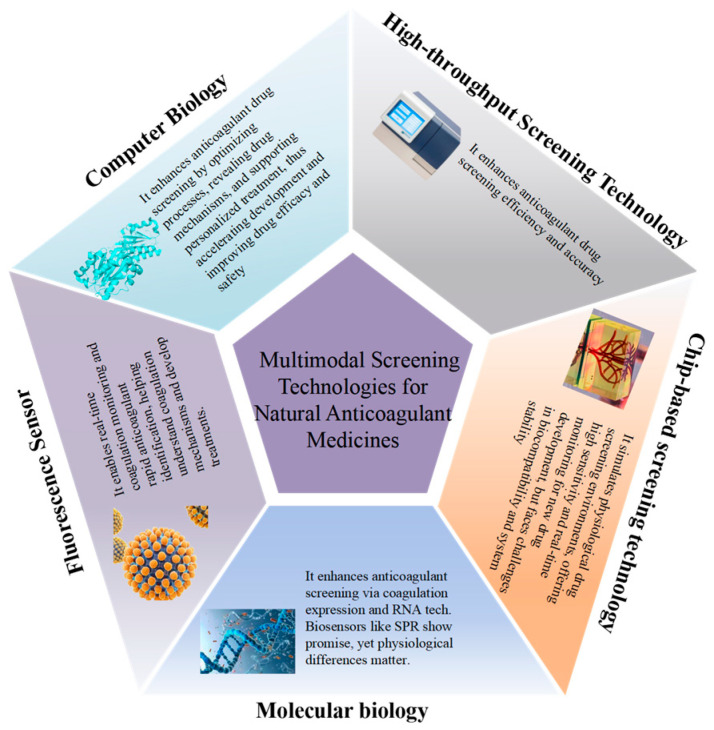
Multimodal screening technologies for natural anticoagulant medicines.

Multimodal screening, with its distinct advantages of precision and efficiency, is emerging as a pivotal technology in the field of medicine development. This article delves into the application of high-throughput screening, chip technology, molecular biology-based screening techniques, fluorescence sensor-based screening, and computational biology in the identification of active components for anticoagulant medicines. As depicted in Figure 2, the employment of these technologies has significantly enhanced the efficiency and accuracy of medicine screening, providing robust technical support for the development of novel anticoagulants. Through these advanced screening methods, researchers can rapidly identify active components with therapeutic potential, thereby accelerating the process of medicine discovery and development.

### 3.1. Based on High-Throughput Screening Technology

In recent years, high-throughput screening (HTS) technology has significantly enhanced the efficiency of in vitro anticoagulant medicine screening. Utilizing automated sample handling, miniaturized reaction systems, and rapid signal detection techniques, HTS combines various detection methods such as fluorescence, luminescence, and chromatography. This enables the efficient assessment of thousands to tens of thousands of compounds within a short period, markedly reducing the screening cycle for candidate medicines. High-throughput screening technology employs automation and parallelization to effectively evaluate the biological activity of large compound libraries, rapidly acquiring vast amounts of data on the structure-activity relationships of candidate compounds. This method significantly enhances the efficiency of medicine discovery and simultaneously improves the reliability of experimental data and the reproducibility of results, providing crucial support for the development of new medicines.

In the field of thrombin activity analysis and inhibitor screening, the development of HTS has provided new tools and methodologies for the research and clinical application of anticoagulant medicines. The portable pH meter photoelectrochemical sensor and its fullerene C60 PEC biosensor developed by Wang [25] and his team can detect thrombin activity through changes in photoelectric current and photovoltage and also provide a sensitive and high-throughput new method for the screening of thrombin inhibitors. Ken [26] and his team used the fluorescent thrombin substrate boc-VPR-MCA for high-throughput blood function determination, which can monitor the coagulation process in real-time under different reaction conditions, thus providing real-time data support for medicine screening. Javier [27] and his team combined biosensors with smartphone photometers, integrating the biosensors into 96-well microplates, and achieving bidirectional screening of thrombin content and inhibitors. These studies not only improve the efficiency and accuracy of thrombin activity analysis but also provide important technical support for the optimization of new medicine development and disease treatment strategies. With these high-throughput screening technologies, researchers can quickly identify and verify potential thrombin inhibitors, thereby accelerating the development process of new medicines.

In the research of active components in natural medicines, the combined application of various technologies has become a common technical strategy. By delving into the activity and mechanisms of action of natural medicine components from different levels, it enhances the efficiency and accuracy of research. Wang [28] and his team utilized spectral effect relationship analysis and liquid chromatography-mass spectrometry (LC-MS) technology to measure the coagulation index of rats in vitro using the SC40 semi-automatic coagulation analyzer, further verifying the significant anticoagulant effects of specific components in natural medicines, such as 15 active components including caffeic acid. Wang [29] and his team employed the biological background method and ultra-high performance liquid chromatography-quadrupole time-of-flight mass spectrometry (UHPLC-Q-TOF/MS) technology to discover that components in Typha angustifolia pollen, including citric acid and linoleic acid, could inhibit thrombin activity. By applying spectral effect relationship analysis, they revealed its potential application value in the identification and analysis of active components in natural medicines. This method relies on the biological activity of thrombin and the combination of chemical markers, providing a novel tool for the evaluation and screening of quality markers of natural medicines components. Peng Jingbo [30] and his team used partial least squares regression, combined with the HPLC fingerprint and coagulation time pharmacodynamic data of Polygonum cuspidatum, to deeply analyze the intrinsic relationship between the two, clarifying that the key indicator for evaluating the quality of natural medicines is the concept of efficacy components. Lü Dong [31] and his team used high-performance liquid chromatography and ultraviolet spectrophotometry techniques to find that compared with hot air-dried powder, fresh Sanqi quick-dried powder and freeze-dried PSanqi powder had increased levels of ginsenoside Rg1, Rb1, total polysaccharides, and total proteins, exhibiting better anticoagulant and analgesic activities. These findings underscore the importance of integrating multiple technologies, which not only enhance our understanding of the active components in natural medicines but also provide a scientific basis for the modern research and medicine development of natural medicines.

Despite the significant advantages of high-throughput screening technology, challenges also exist in its application process. First, due to the complexity of biological systems, a large number of false positives or false negatives may occur [32], necessitating secondary screening or more precise biochemical validation to ensure the accuracy of results [33]. Second, as compound libraries continue to expand, the complexity of data processing and analysis also increases significantly. Additionally, high-throughput screening (HTS) typically targets specific molecular targets, which may overlook the multi-target effects and complex physiological mechanisms of anticoagulant medicines. It might also neglect potential medicines that act through atypical mechanisms, including those that involve new targets [34], new therapeutic pathways, or the exploitation of new mechanisms of existing medicines [35] to achieve therapeutic effects.

### 3.2. Chip-Based Screening Technology

Chip-based screening technology combines microfluidic chips with biosensing techniques, utilizing microfluidic chips [36] to simulate the flow of blood in blood vessels, closely mimicking the medicine screening environment under physiological conditions. Chips corresponding to the type and mechanism of action of the medicines being screened are established and appropriate biosensing and detection techniques are integrated to identify and separate individual cells or molecules [37] for precise evaluation of medicine efficacy. Taemin [38] and his team developed a biosensor composed of a polydimethylsiloxane (PDMS) channel layer on a glass substrate that can perform high-sensitivity real-time detection of thrombin through electrochemical impedance spectroscopy, with a detection range of thrombin concentration from 0.1 to 105 ng/mL. Additionally, the surface-modified ITO glass-PDMS hybrid microfluidic chip developed by Wang [39] and his team, through the integration of fluorescence imaging and MALDI-MS, can monitor and quantify enzyme reactions within the microfluidic chip. This method can rapidly and accurately screen for compounds with thrombin inhibitory activity, such as luteolin, saponins, and baicalin, effectively and efficiently identifying thrombin inhibitors from natural products.

Microfluidic chip technology has significantly improved the sensitivity, repeatability, and portability of detection systems by integrating multiple sensing modules such as optics, electrochemistry, and microwave sensors. This highly integrated sensor system also endows microfluidic chips with the ability to monitor biological effects and biomarker changes in biological samples in real-time. Importantly, this monitoring capability makes it possible to study disease mechanisms, medicine effects, and other biological processes under simulated physiological conditions, thereby providing an accelerated impetus for the development of new medicines and the optimization of disease treatment strategies. Li [40] and their team achieved precise control over the flow of blood samples by constructing a microvascular network on a microfluidic chip, allowing for real-time monitoring of the blood coagulation and dissolution process. This innovative microfluidic chip technology not only simulates the fluid environment within blood vessels and studies the deformation properties of blood cells at different flow rates but also plays a significant role in understanding the pressure changes of cells in the blood circulation and the relationship between cell deformation and diseases. Despite the many advantages that microfluidic chip technology exhibits in the field of medicine screening, such as precise fluid control, reduced reagent consumption, high-throughput experiments, and automation, it still faces some challenges in widespread application, mainly including improving the biocompatibility of the chips, enhancing the stability and repeatability of the system. To overcome these technological bottlenecks, it is necessary to conduct in-depth research on new materials and surface treatment technologies, improve chip design and manufacturing processes, and develop more precise fluid control systems and detection methods. In addition, the mass production of microfluidic chips, quality control, and the cultivation of interdisciplinary talents are also key factors in driving the development of this technology. As these challenges are gradually addressed, microfluidic chip technology has the potential to provide a more efficient and accurate screening platform for the research of natural antithrombotic medicines, thereby accelerating the development of new medicines and the optimization of disease treatment strategies.

### 3.3. Molecular Biology-Based Screening Techniques

Molecular biology techniques utilize methods such as molecular cloning [41], protein engineering [42,43], and gene expression [44,45] to construct expression systems containing key indicators of the coagulation system, thereby rapidly identifying and evaluating the effects of medicines on these indicators. For example, key proteins such as thrombin or plasmin can be recombinantly expressed and purified to establish in vitro enzyme activity detection systems for screening small molecule compounds that can inhibit or activate these enzymes; RNA techniques can be used to silence coagulation-related genes, not only to understand their function in the coagulation process but also to explore their potential as therapeutic targets. Zsuzsa [46] developed a new protein determination method that utilizes FXIII-A transpeptidase activity to achieve high-sensitivity enzyme activity kinetic measurements in complex biological samples. Shi [47] and his team successfully immobilized thrombin on modified amino-silica using high-performance liquid chromatography, providing a new approach for the screening of direct thrombin inhibitors. Wang Yuxin [48] and his team used chromatographic separation techniques combined with nuclear magnetic resonance spectroscopy and mass spectrometry to identify the structures of monomeric compounds in 10 coumarins, validating the potential application value of these compounds through prothrombin time tests. Raja [49] and his team revealed the important role of protein S gene silencing in hemophilia treatment through their research, providing a theoretical basis for the development of future targeted therapies.

Additionally, emerging biosensor technology is gradually being applied to the in vitro screening of anticoagulant medicines. Bai [50] and his team immobilized thiol-modified thrombin aptamer (TBA29) on gold nanoparticles (AuNPs) through Au-S bonds, and developed an aptamer/thrombin/aptamer-AuNPs SPR sensor using biotinylated thrombin aptamer (TBA15) grafted onto streptavidin-precoated SPR gold films, which can detect thrombin in real-time. Rinosh [51] and his team utilized dual-cell SPR technology and thrombin aptamers to detect coagulation factors in plasma samples, demonstrating the application of SPR in establishing highly sensitive, real-time label-free biosensors. Furthermore, Tang [52] and his team used SPR technology to form DNA sequences complementary to the fixed aptamers with the 15-mer aptamers of thrombin, providing a new research direction for the development of DNA aptamer-based biochips and biosensors. These studies demonstrate a deep understanding of the blood coagulation process at various levels and its potential application in the development of therapeutic methods.

However, despite the fact that modern molecular biology techniques have improved the precision and efficiency in the screening of natural antithrombotic medicines, it is necessary to consider the differences between in vitro systems and physiological environments when applying these technologies. The simplification of in vitro experimental conditions may overlook some important physiological factors. Therefore, when interpreting the results of medicine screening, it is necessary to cautiously combine in vitro data with biological significance to ensure scientific validity and clinical relevance.

### 3.4. Fluorescence Sensor-Based Screening Technology

Fluorescence sensor screening technology is a promising field in the development of natural antithrombotic medicines. This technique combines the high sensitivity and selectivity of fluorescence technology, effectively meeting the dual needs of real-time monitoring of the coagulation process and screening of natural antithrombotic medicines. By adding the constructed sensor to the sample to be tested, a rapid response result can be obtained; when anticoagulant substances are added, different fluorescence signals are produced, thereby reflecting the strength of anticoagulant activity, providing a new tool and method for screening anticoagulant active substances. Zhang [53] and his team developed trifunctional protein HTs for a thrombin biosensor, the intensity of whose surface fluorescence signal varies according to the concentration of thrombin, allowing for the flexible application of protein engineering in the screening process of natural antithrombotic medicines. Li and colleagues [54] asynthesized an aggregation-induced emission (AIE) sensor, AIENP, and by integrating it with UHPLC-Q-TOF/MS technology, rapidly identified 58 chemical constituents in Xuanbeijing injection. Among these, six compounds exhibited significant anticoagulant activity. This discovery not only provides a novel, cost-effective, and straightforward method for screening active components in natural medicines but also offers a targeted approach for the study of thrombin inhibitors. Furthermore, Shunsuke [55] and his team constructed a DNA aptamer array by modifying different sites of blood enzymes, utilizing optical fingerprint sensing for rapid high-throughput medicine screening. Zeng [56] and colleagues developed a highly reproducible in-vitro flowing clot lysis platform with real-time fibrinolysis monitoring to screen thrombolytic medicines, demonstrating tPa-dependent thrombolysis both via clot mass loss and fluorometrically monitored release of FITC-labeled fibrin degradation products, thereby showing the influence of pulsatile flow on medicine activity.

The applications of fluorescence probes have not only demonstrated their advantages in terms of rapidity, high sensitivity, and high specificity, but also helped researchers gain a deeper understanding of the mechanisms of coagulation activity, providing a scientific basis for the development of anticoagulation treatment strategies. Furthermore, fluorescence probes can be used to label specific molecules or cells to observe the dynamic process of thrombus formation, aiding in the discovery of new therapeutic targets and strategies. With the advancement of nanotechnology, nanoscale fluorescence probes are more easily localized to specific areas within cells or the bloodstream, achieving precise anticoagulation [57,58], while reducing adverse reactions [59], showing great potential in medicine delivery and targeted release.

### 3.5. Computer Biology-Based Screening Technology

The integrated application of computer-aided design [60,61] and bioinformatics [62,63] heralds a revolutionary transformation in the screening process. The first aspect is the construction of databases and data mining. In vitro natural antithrombotic medicines screening technologies utilize computer biology techniques to conduct large-scale medicine screening and virtual screening through databases such as the medicine-Gene Interaction Database (Dgidb, https://dgidb.org (accessed on 1 January 2024)) [64], the compound database PubChem (https://pubchem.ncbi.nlm.nih.gov (accessed on 1 January 2024)) [65], and the RCSB Protein Data Bank (PDB, https://www.rcsb.org/ (accessed on 1 January 2024)) [66]. These resources enable the prediction of candidate medicines’ potential and efficacy, thereby optimizing the screening process for natural antithrombotic medicines.

Secondly, molecular docking simulation technology can simulate the binding modes and interactions between medicines and target proteins, aiding in the understanding of the medicine’s mechanisms of action and guiding the design and screening of appropriate medicines. Zhu and his team [67] studied 100 individual metabolites derived from traditional Chinese medicine using molecular docking simulation technology, discovering that 51 metabolites exhibited angiogenesis activity and predicted the angiogenesis regulatory activity of the remaining 49 metabolites. Concurrently, computational biology methods can integrate multi-omics data, such as genetic information, gene expression profiles, and metabolomic data from different patients, to support personalized screening and treatment of natural antithrombotic medicines. In cardiovascular disease patients, a computational analysis combining miRNA sequencing with coagulation phenotypes can identify new links related to increased thrombosis risk. Kreutz [68] and his team used miRNA sequencing and computational analysis of coagulation phenotypes to investigate new links between coagulation and miRNA in cardiovascular disease patients. They found that citrate plasma-activated thromboelastography analysis could reveal miRNAs associated with increased thrombosis risk, further validating the association of these miRNAs with coagulation factors or coagulation-related gene targets.

Additionally, computational biology techniques can predict the side effects, interactions, and metabolic pathways of medicines, assess the safety and potential risks of medications, and provide important guidance for rational medicine selection and medication management. Kyle and his team [69] used computational studies to predict the activity of 656 medicines on 73 protein targets associated with adverse medicine reactions, revealing that the abdominal pain side effect caused by the estrogenic chlorotrianisene is mediated through the inhibition of COX-1, and this result was verified through platelet aggregation tests. On the other hand, the application of artificial intelligence technology in pattern recognition [70] and the construction of predictive models [71] provides more accurate and reliable guidance for in vitro natural antithrombotic medicines screening techniques. Yassir and his team [64] conducted structure-based virtual screening and computational pharmacokinetic assessments on 11,648 natural compounds using the CHEMBL dataset, discovering that demethoxycurcumin could serve as a potential medicine for the treatment target outer membrane protein W, and could be used as a monotherapy or in combination with colistin. The application of computational biology and artificial intelligence technologies offers new possibilities for medicine development and screening, promising to accelerate the medicine development process, enhance the efficacy and safety of medicines, and bring more hope for clinical treatments.

When conducting in vitro antithrombotic medicine screening, HTS proves to be highly effective for screening large compound libraries, with its sensitivity and specificity depending on the detection method used. While it demands substantial resources, strategies can be implemented to minimize expenses. Microfluidic chips provide exceptional sensitivity and accuracy for screening at the cellular level, albeit with operational intricacies. Molecular biology techniques offer detailed insights into mechanisms but come with high costs and technical challenges. Computational biology is advantageous for virtual screening and medicine design, being cost-effective but also necessitating specialized knowledge. Each method has its own advantages, and the selection should be based on a balance between research objectives, budget constraints, and practical considerations.

## 4. Limitations and Prospects of In Vitro Screening Techniques

The development of in vitro natural antithrombotic medicines screening techniques has provided a wealth of data and potential candidates for medicine research, with precise interpretation of the screening results being crucial, as it directly affects the direction of medicine candidate development and their prospects for clinical application. In the research and application of in vitro natural antithrombotic medicines screening techniques, accuracy and reproducibility are the core indicators for evaluating the effectiveness of the screening. Accuracy ensures that the screening results truly reflect the activity of natural antithrombotic medicines, while reproducibility pertains to the consistency of the screening results under the same experimental conditions. However, ensuring the stability of these two indicators faces multiple challenges in practical operations, as shown in Table 1.

While in vitro screening methods for natural anticoagulant medicines offer significant advantages, they also exhibit certain limitations when compared to in vivo screening. For instance, although microfluidic chip technology can construct highly precise microvascular models, it still fails to fully simulate the dynamic changes in blood flow and the interactions between tissues and organs within the body. Moreover, the concentration gradients generated by microfluidic chips are highly sensitive to changes in flow rate. Similarly, organoid and fluorescence screening techniques also have their limitations in in vitro models. Organoid models, despite their ability to mimic the structure and function of tissues and organs, still differ in cultivation conditions from the in vivo environment, which may lead to discrepancies in medicine mechanisms and effects. Fluorescence screening techniques, while highly sensitive, are susceptible to interference from background fluorescence and non-specific binding.

To address these limitations, several approaches can be taken: first, optimize the design of microfluidic chips. For example, the high-flow-resistance microchannel network structure designed by Hattori [75], it is not only compact but also capable of separating different concentration gradients, thereby reducing structural complexity and operational difficulty. Second, develop in vitro models that more closely resemble the in vivo environment, such as multicellular co-culture systems, to better simulate the interactions between tissues and organs. Third, establish unified assessment standards and quality control processes for in vitro models to ensure the reproducibility and comparability of results across different research teams. Finally, conduct more in vivo experiments to validate the predictive results of in vitro models, ensuring the effectiveness and reliability of in vitro screening methods.

Natural medicines play a significant role in anticoagulant therapy and have been applied in clinical settings. By screening antithrombotic medicines using the aforementioned techniques, a stronger foundation can be provided for new medicine development. In the future, these technologies will play a greater role in populations at risk of cardiovascular diseases. Microfluidic chip technology, which constructs in vitro microvascular models, can accurately assess the efficacy of antithrombotic medicines and provide personalized treatment plans. Computational biology techniques optimize medicine design through virtual screening and molecular docking, enhancing efficacy and safety.

In the process of in vitro anticoagulant medicine screening, researchers utilize a comprehensive suite of assessment methods to thoroughly evaluate the impact of these components on the blood system. Methods such as cytotoxicity testing [76] and hematological examinations allow for a comprehensive assessment of the effects of natural medicine components on the blood system [77]. Most importantly, natural antithrombotic medicines do not act on a single target but exhibit synergistic effects on multiple targets [78,79]. Existing medicine screening methods often struggle to rapidly and accurately evaluate multiple targets, leading to complexities in bioinformatics. To provide more precise interpretations, it is necessary to integrate knowledge and techniques from various fields such as biology, molecular biology, bioinformatics, pharmacology, and computer science [80], deepening our understanding of medicine mechanisms of action and providing more scientific interpretations of screening results. In practice, researchers must consider the dynamic balance of various factors in anticoagulation screening technology and potential medicine interactions. By precisely analyzing these complex factors and experimental data, researchers can conduct a detailed assessment of the anticoagulant effects of candidate medicines [81,82], effectively screening for new natural antithrombotic medicines with significant clinical application potential. These studies have not only advanced the development of quality assessment and efficacy research methods for natural medicines but also laid a solid foundation for the scientification, standardization, and clinical application of natural medicines. The integration of multimodal and multi-tiered approaches in anticoagulant medicine activity component screening will be more precise and efficient, offering new hope and opportunities for the study of the overall mechanisms of action of natural medicines and the correlation between natural medicines and diseases.

## Figures and Tables

**Table 1:** Limitations of in vitro natural antithrombotic medicines screening techniques.

Limitations		Solution
Experimental Condition Variations	Adjusting temperature, pH, and ionic strength can greatly impact enzyme activity [72]	Minimize adjustments to temperature, pH, and ionic strength
Individual Variations in Biological Materials	Biological materials from different donors may cause inconsistent screening results [73]	Use donors from the same source as much as possible
Simplicity of the Model	Some in vitro models may not fully replicate biological complexity [74]	Complex designs and high tech demands optimize models for biological relevance
Other Factors	Reagent batch variations, operator proficiency, and equipment precision	Use same reagent batch, enhance operator proficiency, and use high-precision equipment
